# The positive role of teachers’ social–emotional competence in burnout: the mediating effects of teaching efficacy

**DOI:** 10.3389/fpsyg.2026.1744311

**Published:** 2026-02-05

**Authors:** Jinqiu Wang, Zaiyun Yang, Xiaohong Chen, Hongxian Chen

**Affiliations:** 1 School of Mathematics and Statistics, Qiannan Normal University for Nationalities, Duyun, China; 2 College of Education Science, Xinjiang Normal University, Urumqi, China; 3 Department of Early Childhood Education, Qiannan Preschool Education College for Nationalities, Guiding, China

**Keywords:** teachers, job burnout, mediation analysis, social–emotional competence, teaching efficacy

## Abstract

**Background:**

Teachers are a high-risk group for job burnout, and alleviating this issue is increasingly urgent. Teachers’ social–emotional competence is closely associated with alleviating occupational burnout and plays a pivotal role in professional development. Consequently, identifying effective methods to reduce teacher burnout has become a central concern in educational science, psychology, and teacher professional development.

**Methods:**

Grounded in the Job Demands–Resources framework, this study developed and tested a mediation model using survey data from 924 Chinese elementary and secondary school teachers. This study delves into the positive role of social–emotional competence in mitigating teacher burnout and elucidates its underlying mechanisms of influence.

**Results:**

Teachers’ social–emotional competence and teaching efficacy negatively predicts teacher burnout. Meanwhile, teaching efficacy mediates the relationship between social–emotional competence and teacher burnout.

**Conclusion:**

Teachers’ social–emotional competence and teaching efficacy, as crucial individual resources, can mitigate teachers’ occupational burnout through dual pathways: “social-emotional competence, occupational burnout” and “social-emotional competence, teaching efficacy, occupational burnout”. This study provides both theoretical insights and practical implications for reducing teachers’ occupational burnout by strengthening teachers’ social–emotional competence and teaching efficacy.

## 1 Introduction

Burnout, a psychological syndrome induced by chronic work-related stress (Maslach et al., 2001), is characterized by the depletion of emotional resources, the emergence of detachment and cynicism toward one’s job, and a decline in feelings of competence and efficacy at work. Teaching is a profession characterized by high levels of exhaustion and emotional fatigue (Hakanen et al., 2006), and it is recognized as one of the most stressful occupations (Johnson et al., 2005). Teachers are required to manage heavy teaching responsibilities and administrative tasks daily (Brante, 2009) and engage with challenging students and parents (Chang, 2013). Compared with many other professions, teachers experience burnout more frequently (Saloviita and Pakarinen, 2021) and are more susceptible to its effects (Squillaci Lanners, 2020). Worldwide statistics reveal that the incidence of burnout among teachers ranges between 30 and 40% (García Carmona et al., 2019), while in China, the burnout rate among primary and secondary school teachers is reported to be 45.5% (Hu et al., 2015). Burnout not only adversely affects the physical and mental well-being of teachers (Salvagioni et al., 2017), but it may also impair work efficiency and teaching quality (Călin et al., 2022), leading to increased turnover (Ingersoll and May, 2012) and indirectly contributing to negative impacts on students’ mental health (Skaalvik and Skaalvik, 2017). This issue is particularly acute in secondary education settings (García Carmona et al., 2019).

Research indicates that cultural, social, and educational conditions contribute to distinct mechanisms underlying the development of teacher burnout (Zhang et al., 2025). Within the Job Demands–Resources (JD-R) model, burnout antecedents are explored across three domains—job demands, job resources, and personal resources (Cheng et al., 2023). Including individual factors such as gender, age, educational background, teaching efficacy, and social–emotional competence (SEC; Lau et al., 2005; Dicke et al., 2014; Zhang et al., 2023; Pikić Jugović et al., 2025), and external factors such as teacher-student relationships (Zhang et al., 2023), leadership style (Onan et al., 2025), and school climate (Yang and Zhou, 2025). Some studies have suggested that individual attributes are the predominant impact on the numerous factors influencing teacher burnout. In contrast, the effect of external factors is relatively weak such as the school environment (Liu et al., 2023) or even negligible (Pas et al., 2012). When changes in the external environment are not feasible in the short term, enhancing internal factors may represent a practical approach to addressing and preventing burnout (Zhang et al., 2023). Against this backdrop, teachers’ personal factors will become the primary drivers of their professional burnout. Teaching activities are both cognitive and emotional endeavors, requiring educators to perceive, interpret, and regulate their own emotions as well as those of their students (Hargreaves, 2001; Li et al., 2025).

Empirical studies indicate that SEC constitutes an antecedent of teacher burnout (McMullin, 2014; Fiorilli et al., 2017; Oliveira et al., 2021; Zhang et al., 2025). Nevertheless, the pathways by which SEC influences teacher burnout remain to be elucidated. This study focuses on the relationship of SEC and teacher burnout, and whether teaching efficacy moderates this relationship. Clarifying these mechanisms will provide practical guidance for mitigating burnout through intrapersonal factors, thereby supporting teachers’ well-being and instructional quality.

## 2 Theoretical framework and literature review

### 2.1 Theoretical framework

The (Yang and Zhou, 2025) is a widely influential framework in occupational health psychology (Bakker et al., 2023), proposing a minimalist structure comprising job demands and job resources. Job demands refer to aspects of work that require sustained physical, cognitive, or emotional exertion, while job resources are elements that facilitate goal attainment, buffer the negative effects of job demands, and promote development (Li et al., 2025). According to the JD-R model, when job demands exceed job resources, stress processes are triggered, depleting energy and ultimately leading to occupational burnout (Bakker and Demerouti, 2017).

Teaching activities possess strong affective attributes, requiring educators to optimize instructional outcomes by regulating their own and students’ emotional states through perception (Hargreaves, 2001). This necessitates teachers possessing strong social–emotional competencies to meet the affective demands of teaching (Savina et al., 2025). Based on the JD-R model, social–emotional competencies serve as the core personal resources enabling teachers to fulfil occupational affective demands (Li et al., 2025). On the one hand, teachers’ social–emotional competence (SEC) is associated with greater emotional clarity (Aldrup et al., 2020). Accordingly, teachers with higher SEC are more adept at recognizing negative emotional triggers underlying stress responses and at employing more constructive cognitive strategies to regulate them (Mérida-López and Extremera, 2017). A study shows that teachers with high SEC may proactively use emotional expression or verbal support to ignite students’ enthusiasm and enjoyment for learning, thus achieving effective classroom management (Jennings and Greenberg, 2009). On the other hand, high social–emotional competence facilitates teacher-student interactions and the establishment of positive relationships, thereby influencing student engagement in the classroom (Schonert-Reichl, 2017). Teachers’ SEC has been identified as a promoter of higher-quality social interactions with principals, colleagues, and children (Garner et al., 2014; Collie, 2017), which would help teachers to obtain more social support to mitigate burnout (Fiorilli et al., 2017).

According to the JD-R model, job resources (including SEC) facilitate work motivation and drive positive outcomes (including reduced burnout). Thus, the JD-R model offers strong applicability and guidance for this study. It provides a meta-theoretical foundation for identifying SEC as a key personal resource and offers a clear framework for examining how this competence mitigates burnout through its motivational mechanisms.

### 2.2 Teachers’ social–emotional competence negatively predicts teacher burnout

SEC, which is grounded in emotional intelligence, may be an antecedent to occupational burnout. Research indicates that emotional intelligence is significantly and negatively associated with burnout (Cofer et al., 2018). Teachers with higher levels of emotional intelligence are more adept at assimilating health-fostering information during emotionally charged situations, enhancing their ability to cope with stress and reducing their burnout (Li et al., 2019). In contrast, when teachers exhibit poor SEC, they often struggle to effectively address the challenges and problems that arise in teaching—a profession inherently stressful. This inability to manage high-stress levels can adversely affect their instructional activities and daily lives, leading to increased occupational burnout (McMullin, 2014). Empirical studies have demonstrated that interventions targeting SEC can alleviate the symptoms of teacher burnout (Oliveira et al., 2021). Drawing on the literature above, this study puts forward the following hypothesis:

*Hypothesis* 1 (H1): Teachers’ social–emotional competence negatively predicts teacher burnout.

### 2.3 Teaching efficacy significantly and negatively predicts teacher occupational burnout

Teaching efficacy mainly refers to teachers’ judgments about their ability to successfully implement instructional behaviors and predict the outcomes that such behaviors may yield. It is primarily manifested in three aspects: instructional process efficacy, classroom management efficacy, and student engagement efficacy (Moran and Hoy, 2001). Teaching efficacy, as a positive personal resource, can assist teachers in managing workplace demands and offset resource depletion (Evertson and Weinstein, 2006). Previous studies have demonstrated the positive role of teaching efficacy in alleviating occupational stress and reducing burnout (Klassen and Chiu, 2010). Teaching efficacy is regarded either as a factor counteracting occupational burnout (Cherniss, 2017) or as a personal resource that alleviates burnout (Dicke et al., 2014). The correlations between teaching efficacy and the three dimensions of occupational burnout range from −0.1 to −0.5 (Brown, 2012), indicating that teachers with high teaching efficacy are more inclined to respond positively to challenges and reduce perceived stress through effective instructional strategies (Klassen and Chiu, 2010). Moreover, teaching efficacy is significantly correlated with occupational burnout, with teachers displaying higher levels of teaching efficacy experiencing lower levels of burnout (Aloe et al., 2014). Based on the aforementioned literature, the present study proposes the following hypothesis:

*Hypothesis* 2 (H2): Teaching efficacy significantly and negatively predicts teacher occupational burnout.

### 2.4 The mediating roles of teaching efficacy

Self-efficacy is regarded as a key internal resource for coping with obstacles and challenges, and the ability to understand and regulate one’s own emotional states is central to its development (Bandura, 1977). For teachers, this form of self-efficacy is mainly reflected in teaching efficacy, which comprises instructional process efficacy, classroom management efficacy, and student engagement efficacy. When performing instructional tasks—such as regulating disruptive student behavior and managing classrooms—teachers must demonstrate proficient emotional regulation, a core component of SEC (Zhang et al., 2025). Evidence indicates that SEC training enables teachers to better perceive and respond to students’ emotional needs and behavioral expressions, thereby supporting the establishment of positive classroom relationships and the implementation of instructional strategies, and ultimately enhancing teaching efficacy (Domitrovich et al., 2016). Specifically, teachers with high SEC can employ proactive emotional expression and verbal support to stimulate students’ motivation and enjoyment, yielding more effective classroom management (Jennings and Greenberg, 2009). Moreover, higher SEC levels promote healthier teacher–student interactions and positive relationships, further increasing student engagement and strengthening teaching efficacy (Schonert-Reichl, 2017). Teachers with higher teaching efficacy tend to perceive work-related stressors as challenges rather than threats, and consequently report lower emotional exhaustion and cynicism; by contrast, teachers with lower self-efficacy are more prone to emotional fatigue and disengagement (Skaalvik and Skaalvik, 2010; Maslach and Leiter, 2016) Existing work indicates negative associations between SEC and teacher burnout, and between teaching efficacy and burnout (Skaalvik and Skaalvik, 2007; Brackett et al., 2010), whereas SEC and teaching efficacy are positively related (Pikić Jugović et al., 2025). In the context of kindergarten education, teaching efficacy mediates the relationship between SEC and burnout (Zhang et al., 2025). Taken together, these findings suggest that teaching efficacy could mediate the SEC–burnout pathway among teachers across primary and secondary education: teachers with high SEC are more likely to display effective instructional behaviors, enhance classroom management and teacher–student interactions, reduce emotional exhaustion and cynicism, and thereby lower the risk of occupational burnout. Based on the existing literature, the present study proposes the following hypotheses:

*Hypothesis* 3 (H3): Teaching efficacy mediates the relationship between social–emotional competence and teacher burnout.

## 3 Methods

### 3.1 Participants

This study employed A random sampling approach to recruit teachers from elementary, middle, and high schools in Guizhou Province. Throughout the data collection process, ethical principles were strictly observed. All teachers were required to complete the survey anonymously and had the option to withdraw at any time. Participants were also informed that the survey information would be used solely for research purposes and that their personal information would be rigorously protected.

A total of 1,015 questionnaires were distributed and subsequently retrieved. After eliminating invalid responses—such as those exhibiting patterned answering and incorrect responses on lie detection items—the final sample comprised 924 valid questionnaires, yielding an effective response rate of 91.03%. Among the 924 teachers, 402 were males (43.5%) and 522 were females (56.5%). In terms of educational level taught, the sample included 360 primary school teachers (39%), 384 middle school teachers (41.5%), and 180 high school teachers (19.5%). The age of the teachers ranged from 22 to 54 years, with 94.8% falling within this bracket. Regarding educational qualifications, 848 teachers (91.7%) held a bachelor’s degree, while 76 teachers (8.3%) possessed a master’s degree or higher. Regarding location, 545 teachers (58.9%) work in urban areas and 379 teachers (41.1%) work in rural areas.

### 3.2 Measures

Teacher’ SEC was related by teachers using 22 items taken from Li (2019). The item includes six dimensions, self-awareness, self-management, others-awareness, others-management, collective-awareness, and collective-management. Higher scores indicating higher social-emotional competence. The Cronbach’s *α* was 0.926, and the validation factor (χ^2^/df = 3.731, RMSEA = 0.064, CFI = 0.947, TLI = 0.947) reached acceptable levels.

Teacher efficacy was measured using the Chinese scale developed by Moran and Hoy (2001). The scale comprises 12 items across three dimensions: classroom management, instruction, and student engagement. Responses using a 5-point Likert scale, where one indicates “does not apply” and five indicates “applies,” with higher scores reflecting a higher level of teacher efficacy. In the present study, the scale demonstrated excellent internal consistency, with a Cronbach’s α of 0.931. Moreover, confirmatory factor analysis yielded acceptable model fit indices (χ^2^/df = 5.274, RMSEA = 0.08, CFI = 0.959, TLI = 0.946), indicating that the scale possesses robust construct validity.

The Chinese version of the Maslach Burnout Inventory was employed to assess teacher burnout (MBI-GS; Maslach and Jackson, 1981). The scale encompasses low personal accomplishment, emotional exhaustion, and depersonalization. Responses were measured on a 5-point Likert scale, with higher scores reflecting more severe burnout. The scale demonstrated adequate internal consistency in this study, with a Cronbach’s α of 0.896. Furthermore, confirmatory factor analysis indicated a satisfactory model fit (χ^2^/df = 5.023, RMSEA = 0.078, CFI = 0.948, TLI = 0.948), thereby confirming the scale’s robust construct validity.

### 3.3 Data analysis

For statistical processing, we used SPSS 25.0 for descriptive statistical analysis, correlation analysis, and reliability testing, and AMOS 21.0 to test the validity of the measurement instruments. We used the Hayes’ PROCESS 3.5 plugin (Bolin, 2014) to examine the mediating effect of teaching efficacy on the relationship between SEC and teacher burnout.

## 4 Results

The results consist of two phases. In the first phase, we perform the Common Method Biases test, discriminant validity test, and correlation analysis to confirm that the data meet the prerequisites for subsequent hypothesis testing. In the second phase, we conduct hypothesis testing. During this phase, we employ the SPSS macro program recommended by Preacher and Hayes and carry out bootstrapping tests to analyze and verify the mediating effects (Preacher and Hayes, 2008). Specifically, we repeatedly take 5,000 bootstrap samples to estimate the indirect effects of the independent variables and generate the confidence intervals (CI) for the results.

### 4.1 Common method deviation test

To reduce common method bias, we primarily implement procedural and statistical controls. For procedural control, we include lie detection items in the questionnaire and randomize the order of the scale items. For statistical control, we perform confirmatory factor analysis (CFA) for statistical power to assess standard method bias (Williams and O'Boyle, 2015). The CFA results show a poor model fit (χ^2^/df = 27.942, RMSEA = 0.171, CFI = 0.816, TLI = 0.775), which indicates that our survey data do not suffer from significant common method bias.

### 4.2 Difference test

This study examined differences in teacher burnout across various groups, as presented in Table 1. First, there was no significant difference in burnout between male and female teachers (*t* = −0.696, *p* = 0.532). Second, an important difference was found according to teaching experience (*F* = 9.818, *p* = 0.000): overall, teachers with longer teaching experience exhibited lower levels of burnout. Third, significant differences were observed in burnout between different education levels (*t* = −2.009, *p* = 0.045), with teachers holding a master’s degree or higher showing significantly higher burnout than those with a bachelor’s degree. Fourth, no significant differences in burnout were identified among elementary, middle, and high school teachers (*F* = 2.069, *p* = 0.127). Finally, the analysis revealed no significant differences in burnout between urban and rural teachers (*t* = 1.41, *p* = 0.159).

**Table 1:** Difference analysis (*N* = 924).

Variable	Type	M	t	*p*	Variable	Type	M	F	*p*
Gender	Male	2.51	−0.696	0.532	Educational stage	primary school	2.47	2.069	0.127
	Female	2.48				middle school	2.57		
						high school	2.55		
region	City	2.56	1.41	0.159	Teaching years	≤5 years	2.67	9.818***	0.000
	Rural	2.49				5-10 years	2.54		
Educational background	Bachelor’s Degree	2.51	2.009*	0.045		10-20 years	2.44		
	Master’s degree or above	2.69				≥20 years	2.39		

*means that *p*-value is < 0.05; ***means that *p*-value is < 0.001.

### 4.3 Correlation and descriptive test

This study examined the interrelationships among the three primary variables. According to Table 2, teaching efficacy was found to be significantly positively correlated with SEC (*r* = 0.762, *p* < 0.01) and significantly negatively correlated with teacher burnout (*r* = −0.341, *p* < 0.01). In addition, SEC exhibited a significant negative correlation with teacher burnout. These significant correlations among the main variables indicate that further mediation analysis is warranted to explore the potential mediating effects (Baron and Kenny, 1986).

**Table 2:** Descriptive and correlation analysis (*N* = 924).

Variable	M	SD	Teaching efficacy	social-emotional competence	Teacher burnout
Teaching efficacy	3.776	0.698	—		
Social–emotional competence	3.892	0.599	0.762**	—	
Teacher burnout	2.531	0.697	−0.341**	−0.344**	—

**means that *p*-value is < 0.01; ***means that *p*-value is < 0.001.

### 4.4 Hypothese test

To carry out hypothesis testing, we constructed a mediation model with teacher SEC as the independent variable, job burnout as the dependent variable, and teaching efficacy as mediating variables. Control variables (gender, teaching years, educational background, and region) were also included. We conducted mediation analysis using PROCESS 3.5, the results showed that SEC significantly negatively predicted teacher burnout (*β* = −0.235, *t* = −4.298, *p* < 0.001, 95% CI = [−0.343, −0.128]). Furthermore, SEC significantly positively predicted teaching efficacy (*β* = 0.885, *t* = 35.682, *p* < 0.001, 95% CI = [0.374, 0.934]), and teaching efficacy significantly negatively predicted teacher burnout (*β* = −0.178, *t* = −3.778, *p* < 0.001, 95% CI = [−0.270, −0.086]), as shown in Table 3. In summary, Hypotheses H1 and H2 were supported, indicating that both teachers’ SEC and teaching efficacy significantly negatively predict teacher burnout, while teachers’ SEC significantly and positively predicts their teaching efficacy.

**Table 3:** Regression estimation results of mediating effects (*N* = 924).

Regression model		Model fitting index			Effect size and significance		Bias-corrected 95% CI	
Dependent variable	Independent variable	R	R^2^	F	β	t	LLCI	ULCI
Teaching efficacy	Gender	0.765	0.585	258.887***	−0.042	−1.363	−0.0104	0.019
	Educational background				−0.114	−2.010*	−0.226	−0.003
	Teaching years				−0.001	0.842	−0.002	0.004
	Region				−0.012	−0.390	−0.073	0.049
	Social–emotional competence				0.885	35.682***	0.837	0.934
Teacher burnout	Gender	0.391	0.153	27.555***	−0.041	−0.912	−0.128	0.047
	Educational background				0.016	0.198	−0.143	0.176
	Teaching years				−0.008	−4.266***	−0.012	−0.005
	Region				−0.039	−0.875	0.125	0.048
	Social–emotional competence				−0.235	−4.298***	−0.343	−0.128
	Teaching efficacy				−0.178	−3.778***	−0.270	−0.086

∗ means that *p*-value is < 0.05; ∗∗∗ means that *p*-value is < 0.001.

**Figure 1:**
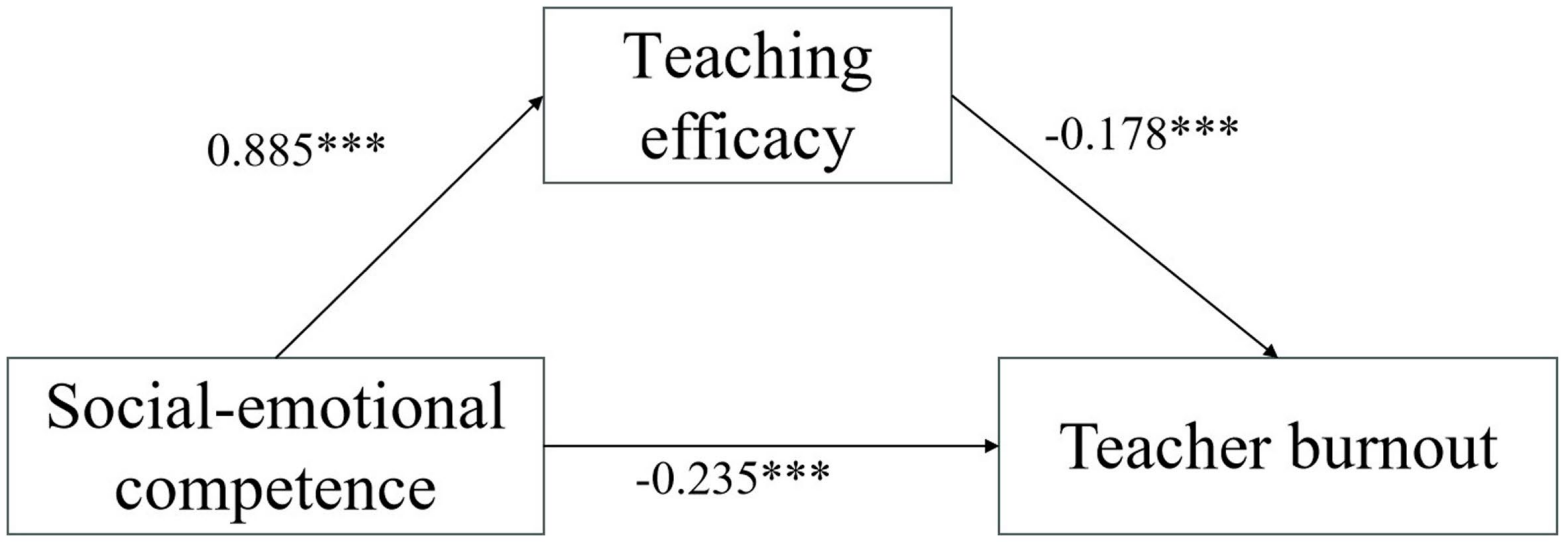
The mediating effect of teaching effectiveness.

Based on Figure 1 and Table 4, the 95% confidence intervals for the direct effect on teacher burnout and the mediating effect of teaching efficacy—as assessed via the bootstrap test—did not include zero. This indicates that the direct effect of SEC on teacher burnout is significant, as is the mediating effect of teaching efficacy. Specifically, the direct effect size was −0.235, whereas the mediating effect size was −0.158, accounting for 58.98 and 41.02% of the total effect, respectively. These findings support Hypothesis H3 by demonstrating that teaching efficacy mediates the relationship between social–emotional competence and teacher burnout.

**Table 4:** Bootstrapping tests.

Path	Effect value	*p*-value	Effect size	LLCI	ULCI
Direct effect	−0.235	<0.01	58.98%	−0.343	−0.128
Indirect effect	−0.158	<0.01	41.02%	−0.242	−0.072
Total effect	−0.393		100%	−0.463	−0.323

## 5 Discussion

### 5.1 The demographic differences in teacher burnout

Regarding gender differences, some studies have found that female teachers experience higher levels of burnout than their male counterparts (Lau et al., 2005), while other research has reported no significant differences in burnout between male and female teachers (Luk et al., 2010). The present study corroborates the latter finding, revealing no significant gender differences in teacher burnout.

Concerning educational attainment, teachers holding a master’s degree or higher exhibit significantly higher burnout levels than those with a bachelor’s degree, a finding consistent with previous studies (Oliveira et al., 2021). There are several possible explanations for this outcome. On the one hand, teachers with higher education levels may have elevated expectations regarding their professional and personal lives. When these expectations are not met in practice, they may experience increased stress and burnout (Maslach and Jackson, 1985). On the other hand, higher-educated teachers face more complex tasks. In contrast, teachers with lower qualifications, due to limited training and experience, may require additional support from their higher-educated peers. These factors could contribute to higher burnout among teachers with advanced degrees (Ozoemena et al., 2021).

The current research indicates no significant differences in burnout levels between elementary and secondary school teachers. This finding can be attributed to several factors. First, within the Chinese educational context, the promotion mechanisms for both elementary and secondary school teachers are similar. Second, in traditional Chinese culture, teaching is regarded as a respected profession regardless of whether one teaches at the elementary or secondary level (Luk et al., 2010).

Regarding geographical differences, regional characteristics affect teacher burnout to some extent. Previous research has documented that urban teachers exhibit higher burnout than their rural counterparts (Abel and Sewell, 1999). However, our findings reveal no significant difference between the burnout levels of urban and rural teachers. This result may be due to the rapid narrowing of the gap between urban and rural areas in China, or it could be related to the relatively small urban–rural differences in the regions represented in our sample (Wu and Zheng, 2012).

Finally, concerning teaching experience, the data indicate that teachers with longer teaching careers display lower levels of burnout compared to those with fewer years of experience, a finding that aligns with previous research (Lau et al., 2005). Several explanations may account for this observation. First, younger teachers may lack the life experience—encompassing interactions with students, parents, colleagues, and supervisors—crucial for managing work-related stress. Second, more experienced teachers are generally better equipped to identify and utilize effective coping strategies to manage stress (Mendes, 2002).

### 5.2 The relationship among teachers’ social and emotional abilities, teaching effectiveness and teacher burnout

Research has revealed that teachers’ SEC is significantly negatively correlated with occupational burnout, serving as a significant negative predictor of burnout. In other words, the higher a teacher’s SEC, the lower their level of occupational burnout. This finding supports Hypothesis 1 and is consistent with previous studies (Oliveira et al., 2021; Tian et al., 2021; Liu et al., 2023; Zhang et al., 2023; Pikić Jugović et al., 2025).

Similarly, teaching efficacy was significantly negatively correlated with burnout and emerged as a significant negative predictor of occupational burnout. This indicates that teachers with higher teaching efficacy experience lower levels of burnout. Hypothesis 2 is validated, aligning with earlier research (Sarıçam and Sakız, 2014; Zhu et al., 2018). One plausible explanation for this result is that teachers with high teaching efficacy are more inclined to proactively address challenges by employing effective instructional strategies that reduce perceived stress (Klassen and Chiu, 2010). Additionally, teaching efficacy influences an individual’s goals and behaviors, with efficacy beliefs shaping how one perceives environmental opportunities and obstacles (Skaalvik and Skaalvik, 2010).

Furthermore, our study found that SEC is significantly positively correlated with teaching efficacy, indicating that teachers with higher SEC tend to exhibit greater teaching efficacy. This is consistent with previous findings (Pikić Jugović et al., 2025). Teachers with strong SEC may be more effective in classroom management and fostering student engagement. Several factors may account for this association. First, teachers with high SEC are more likely to adopt efficient teaching strategies (Koçoğlu, 2011). Second, such teachers might actively utilize emotional expression or verbal support to ignite students’ passion for learning, thereby enhancing classroom management (Jennings and Greenberg, 2009). Third, educators with elevated SEC are better equipped to facilitate positive teacher-student interactions and construct a supportive classroom environment, promoting students’ engagement in learning (Schonert-Reichl, 2017).

### 5.3 The mediating mechanism of teaching efficacy

Research has found that SEC, teaching efficacy, and occupational burnout are significantly negatively correlated, with teaching efficacy serving as a mediator between SEC and occupational burnout, thereby confirming research hypothesis H3. The JD-R model posits that factors influencing occupational burnout can be classified into job demands and resources (Demerouti et al., 2001). For teachers, job demands include workload, role stress, emotional demands, maladaptive student behaviors, promotion pressure, and professional ethics. In contrast, job resources encompass organizational support, colleague support, infrastructural conditions, autonomy, job meaning, and rewards (Wu et al., 2014). Teacher’s SEC is closely associated with role stress, emotional demands, and maladaptive student behaviors. Teachers with high SEC can better regulate their emotions in the service of their instructional tasks, which allows them to secure more favorable student feedback and enhance their teaching efficacy (Jennings and Greenberg, 2009). Moreover, such teachers tend to establish positive teacher-student relationships more effectively, facilitating the management of maladaptive student behaviors and increasing student engagement, thereby further improving teaching efficacy (Schonert-Reichl, 2017) and ultimately reducing occupational burnout.

An increasing number of scholars have incorporated personal resources into the JD-R model (Xanthopoulou et al., 2009), and evidence suggests that personal resources such as self-efficacy and optimism mediate the influence of job resources on occupational burnout (Huang et al., 2016). Teachers’ SEC and teaching efficacy are important personal resources (Evertson and Weinstein, 2006; Zhang et al., 2023). Teachers with high SEC are more likely to garner greater organizational and colleague support, which in turn facilitates an increase in teaching efficacy and a reduction in occupational burnout (Moran and Hoy, 2001). This study further explored the impact of SEC and teaching efficacy on occupational burnout, finding that the direct effect of SEC on burnout accounted for 58.98% of the total effect, while the indirect effect, mediated through teaching efficacy, accounted for 41.02%. This finding suggests that teachers’ SEC exerts a substantial and direct influence on alleviating burnout, reflecting its fundamental role in emotional regulation and stress management. From a practical perspective, these results suggest that, under constrained resources, prioritizing training designed to enhance teachers’ SEC may lead to more rapid improvements in emotional states and mental health. Nonetheless, given the importance of teaching efficacy in promoting a positive educational environment, a combined enhancement of both factors may offer beneficial trajectories for teachers’ long-term professional development.

### 5.4 Contributions and limitations

At the theoretical level, this study first expands research on teachers’ social-emotional competence, teaching efficacy, and professional burnout. Previous studies have predominantly examined the impact of individual factors on teacher burnout, focusing primarily on the effects of self-efficacy, classroom disruptions (Dicke et al., 2014; Dicke et al., 2014; Wang et al., 2015; Cherniss, 2017), and leadership styles (Tian et al., 2021; Onan et al., 2025). By establishing a direct link between social-emotional competence and teaching efficacy/burnout, this research highlights the unique influence mechanism of social-emotional competence on burnout. Secondly, the study enriches the application of the JD-R model within teacher professional development. It demonstrates that personal core resources can mitigate teacher burnout through the pathways “social-emotional competence – burnout” and “social-emotional competence – teaching efficacy – burnout”, thereby validating and extending the theoretical propositions of the JD-R model (Bakker and Demerouti, 2017). Finally, the study proposes a mediation effect model constructed through teaching efficacy, suggesting that reducing teachers’ occupational burnout can be achieved by simultaneously enhancing psychological capital, such as their social-emotional competence and teaching efficacy.

Practically, this research offers actionable pathways for teacher professional development and school management. Firstly, teacher training should strengthen social-emotional learning through systematic courses or workshops on emotion management and regulation, enhancing educators’ capacity to regulate their own and students’ emotional states and foster psychologically supportive classroom environments. Secondly, educational administrators should cultivate a harmonious work atmosphere, ensuring teachers possess adequate resources to fulfil their teaching duties through the provision of emotional support and peer assistance. Finally, teachers can develop emotional management capabilities through self-directed learning and reflective practice. By employing positive emotional states and emotional regulation strategies in teaching, educators can manage both their own and their students’ emotions, thereby enhancing teaching efficacy and alleviating professional burnout.

Several limitations have been identified based on the entire research process. First, this study falls within cross-sectional research; thus, the findings cannot be used to infer causal relationships. Future studies should adopt longitudinal or experimental designs to explore the causal relationship between SEC and teachers’ occupational burnout. Although the present study demonstrated that SEC and teaching efficacy significantly predict occupational burnout and teaching efficacy is a mediator between SEC and burnout, SEC—as an antecedent of burnout—may also influence occupational burnout through other mediating variables. Therefore, future researchers are encouraged to devote greater attention to the potential positive effects of SEC on occupational burnout, to uncover the underlying mechanisms, and to identify more diversified and effective strategies for reducing teachers’ occupational burnout. Moreover, SEC, teaching efficacy, and burnout have been treated as unidimensional constructs, with limited consideration of their potential dimensions. Future research should examine the latent dimensionality of these variables in greater detail to provide a more nuanced understanding of their interrelations.

## 6 Conclusion

The study found that both SEC and teaching efficacy significantly and negatively predict teacher burnout, while SEC significantly and positively predicts teaching efficacy. Moreover, teaching efficacy mediates the relationship between SEC and teacher burnout. Among the demographic variables examined, no significant differences in teacher burnout were observed based on gender or between urban and rural teachers, and there were no significant burnout differences among primary, middle, and high school teachers. Significant differences in teacher burnout were observed only concerning the educational background and teaching years.

## Data Availability

The original contributions presented in the study are included in the article/supplementary material, further inquiries can be directed to the corresponding author.
